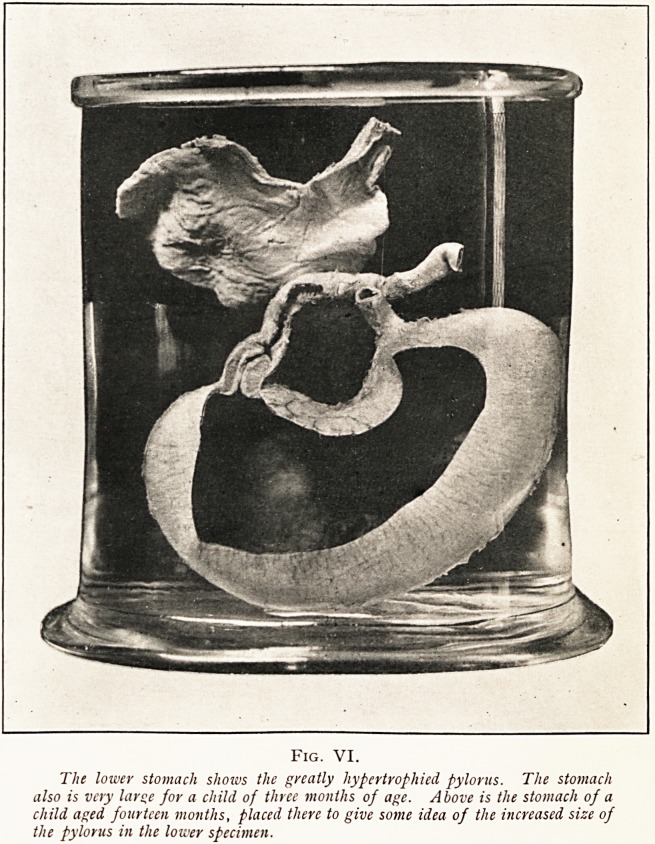# A Case of Congenital Hypertrophic Stenosis of the Pylorus

**Published:** 1904-06

**Authors:** Theodore Fisher, Newman Neild

**Affiliations:** Physician to Out-patients, Bristol Hospital for Sick Children; Pathologist to the Bristol Royal Infirmary; Assistant Physician and Pathologist to the Bristol General Hospital


### A CASE OF CONGENITAL HYPERTROPHIC STENOSIS OF THE PYLORUS.

BY

THEODORE FISHER, M.D.Lond., M.R.C.P.

*(Physician to Out-patients, Bristol Hospital for Sick Children; Pathologist to the Bristol Royal Infirmary)*,

AND

NEWMAN NEILD, M.B., Ch.B.Vict.

*(Assistant Physician and Pathologist to the Bristol General Hospital)*.

---

CASES of congenital hypertrophic stenosis of the pylorus have been so frequently noticed of late that the mere record of a case is of little or no interest. Possibly, however, the accompanying photographs justify the publication of a few brief notes of an example of this disease.

Early in January of this year a male infant, aged eight weeks, was seen in the out-patient department of the Bristol Hospital for Sick Children, having been brought there by its mother for vomiting and wasting. As is said to be usually the case with infants in which there is congenital stenosis of the pylorus, the child was described as a "beautiful baby born." Apparently it retained the milk in the stomach during the first three weeks of life, at the end of which time vomiting commenced. When seen at the Children's Hospital the child was considerably wasted, and on palpation of the abdomen a small mass could be felt just below the right costal margin near the external border of the rectus muscle, which it was thought might be a hypertrophied pylorus. No peristalsis of the stomach could, however, be elicited. Further questioning of the mother brought out the fact that the child did not always vomit immediately after taking food, sometimes not until an hour had elapsed, or even at comparatively long intervals during the day—that is to say, vomiting did not necessarily follow every feed of milk. The mother refused to allow the child to remain in the hospital, but three weeks later returned with the infant, remarking that she had noticed —what she had been told to look for—occasional swelling in the epigastrium. Almost immediately the abdomen was uncovered the truth of her observation became obvious. A swelling the size of a hen's egg appeared, which travelled from the left costal margin to the external border of the right rectus muscle.

Two or three days after admission photographs were taken of the infant's abdomen during peristalsis. Unfortunately the day was dull and rainy, yet with exposures of a quarter of a second photographs were obtained in the ward which give a good idea of some of the appearances of the epigastrium during peristalsis of the stomach. It would have been better perhaps had we obtained a photograph also of the epigastrium when the stomach was at rest. There was then no fulness whatever in the epigastrium.

Fig. I.

*Two waves of peristalsis.*

Fig. II.
*Swelling near the left costal margin, due to partial contraction of the stomach.*

Fig. III.
*The greater part of the stomach standing out, owing to muscular contraction.*

Fig. IV.
*Rather more of the stomach brought into prominence.*

Fig. V.
*Peristalsis passing towards the pylorus.*

Fig. VI.

*The lower stomach shows the greatly hypertrophied pylorus. The stomach also is very large for a child of three months of age. Above is the stomach of a child aged fourteen months, placed there to give some idea of the increased size of the pylorus in the lower specimen.*

The child was obviously in too weak a condition for operation, and although nutrient enemata were administered and sterilised oil was injected under the skin, the emaciation continued, and death took place during the twelfth week of life.

On the day before death peristalsis had ceased, but the stomach was then, curiously enough, always clearly visible and much larger than before, extending about one inch below the umbilicus. At the time of the autopsy it was larger still, and occupied the whole abdomen. The intestines throughout were collapsed and contained no fæces.

A reference to the accompanying photograph will give a better idea of the appearance of the stomach than a written description. The greatly hypertrophied pylorus is clearly seen. For comparison the stomach of a much older child, a girl aged fourteen months, is placed above the specimen. It will be noticed that the pylorus in the child of three months of age is much larger than that of a child nearly five times as old. The comparative size of the two stomachs, however, should be neglected. The larger stomach was distended when fresh, found to contain fourteen ounces, and was hardened when full of the fluid. The smaller stomach, in which it may be remarked are tuberculous ulcers, has been laid open, and in its collapsed condition the hardening fluid in which it was preserved has contracted it. Had both stomachs been distended before being hardened they would have been more nearly of the same size; but fourteen ounces is considerably above the average capacity of a stomach of a child of fourteen months, so that even when distended the stomach of the older child would still have been the smaller.

We are indebted to Dr. Fortescue Brickdale for the photographs here reproduced.

## Figures and Tables

**Fig. I. Fig. II. Fig. III. Fig. IV. Fig. V. f1:**
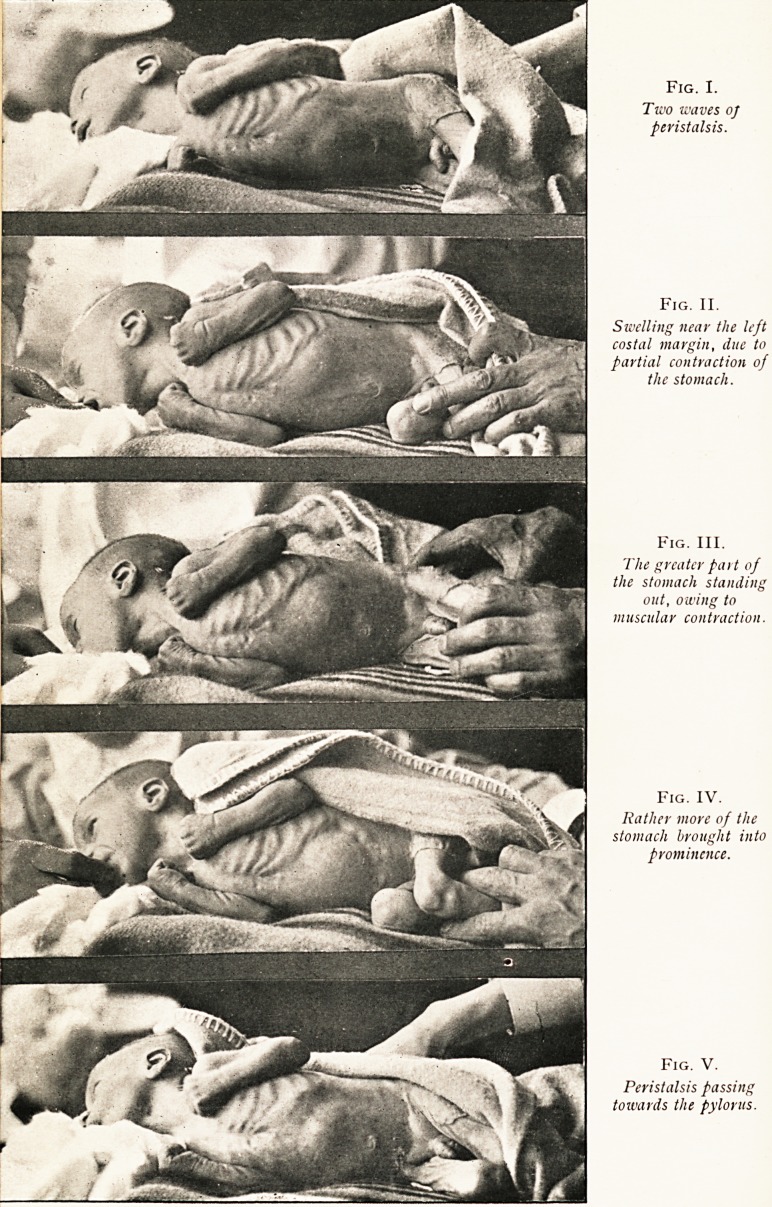


**Fig. VI. f2:**